# Supplementary material for: Blood Lead (Pb) Levels as a Possible Marker of Cancer Risk in a Prospective Cohort of Women with Non-Occupational Exposure
Source: Biomedicines. 2025 Jun 29;13(7):1587. doi: 10.3390/biomedicines13071587 (PMC12292227; doi:10.3390/biomedicines13071587)
Supplement: Supplementary file 1 [file biomedicines-13-01587-s001.zip › biomedicines-3651953-supplementary.pdf]

Table S1 Hazard ratios for any cancer by lead level for group age>=50 (quartiles).

			Univariate COX Regression			Multivariate COX Regression *		
**Pb level** µg/L	**Cases**	**Unaffected**	**HR**	**95% CI**	***p*-value**	**HR**	**95% CI**	***p*-value**
Q1 2.58-9.39	21(10.4%)	180(89.6%)	—	—	—	—	—	—
Q2 9.4-12.58	37(9.5%)	350(91.5%)	0.83	0.48-1.42	0.5	0.823	0.48-1.41	0,48
Q3 12.59-17.17	54(10.3%)	468(89.7%)	0.84	0.51-1.4	0.52	0.826	0.49-1.37	0,46
Q4 17.18-96.27	59(9.8%)	541(91.2%)	0.77	0.46-1.27	0.31	0.72	0.43-1.20	0.20
Q1 2.58-9.39 vs Rest 9.4-96.27	150(9.9%)	1359(90.1%)	0.8	0.51-1.28	0.37	0.78	0.49-1.24	0.30

***(smoking. 1st degree relatives. adnexectomy. oral contraception. hormone replacement therapy. arsenic quartiles level. cadmium quartiles level)**

Table S2 Hazard ratios for breast cancer by lead level for group age>=50 (quartiles).

			Univariate COX Regression			Multivariate COX Regression *		
**Pb level** µg/L	**Cases**	**Unaffected**	**HR**	**95% CI**	***p*-value**	**HR**	**95% CI**	***p*-value**
Q1 2.58-9.39	9(4.7%)	180(95.3%)	—	—	—	—	—	—
Q2 9.4-12.58	12(3.3%)	350(96.7%)	0.63	0.26-1.50	0.30	0.58	0.24-1.39	0.22

Q3 12.59-17.17	22(4.4%)	468(95.6%)	0.80	0.36-1.74	0.58	0.73	0.33-1.61	0.448
Q4 17.18-96.27	31(5.4%)	541(94.6%)	0.93	0.44-1.97	0.86	0.77	0.36-1.65	0.51
Q1 2.58-9.39 vs Rest 9.4-96.27	65(4.5%)	1359(95.5%)	0.81	0.40-1.64	0.57	0.716	0,35-1,45	0.35

***(smoking. 1st degree relatives. adnexectomy. oral contraception. hormone replacement therapy. arsenic quartiles level. cadmium quartiles level)**

Table S3 Other studies results

N	Type of study group	Type of Cancer	Results<br>Q1 or tertile 1 reference	References
EPIC-Italy 47749<br>NSHDS 95000	Populational	B-cell NHL and Multiple myeloma<br>Total cases194	Q4 (87.531-400.843 µg/L)OR 0.93;CI95%(0.43-2.02);p 0.852	[1]

The Finnish cohort 20.752<br>The Great Britain cohort 9122	Occupational exposure	Total cases 7000	Q1<20µg/dL<br>Q2 20-29 µg/dL<br>Q3 30-39 µg/dL<br>Q4 40+ µg/dL	[2]
		Brain malignant	Q4HR1.71;CI95%(0.94-3.12);p 0.04	
		Glioma	Q4HR1.4;CI95%(0.71-2.76);p 0.05	
		Meningioma	Q3HR2.16;CI95%(0.66-7.07);p 0.35	
		Oesophagus	Q3HR2.00;CI95%(1.08-3.71;p 0.009	
		Hodgkin's lymphoma	Q3HR2.37;CI95%(0.87-6.43);p 0.15	
		Lung	Q4HR1.57;CI95%(1.30-1.90); p<0.0001	
		Melanoma	Q2HR1.09;CI95%(0.8-1.48);p 0.95	
		Rectum	Q4HR1.49;CI95%(1.03-2.17);p 0.55	
		Stomach	Q2HR1.55;CI95%(1.10-2.18);p 0.84	

		Kidney	Q2HR1.05;CI95%(0.75-1.48);p 0.51	
		Bladder	Q4HR1.24;CI95%(0.87-1.75);p 0.38	
		Larynx	Q4HR1.92;CI95%(0.94-3.91);p 0.13	
NHANES 16034	Populational	Total cases 202 Skin cancer	Q4(0.11μg/dL)OR 0.71;CI95%(0.45–1.11);p 0.481	[3]
NHANES 94337	Populational	Cases788 Breast cancer	OR0.83;CI95%[0.75. 0.92]; p <0.001	[4]
		Cases 113 Ovarian Cancer	OR1.06;CI95%[1.00- 1.13];p 0.044	
		Cases784 Prostate Cancer	OR1.08;CI95%[1.04- 1.11]; p < 0.001	
		Cases 33 Testicular cancer	OR1.06;CI95%[0.97. 1.16]; p 0.618	

CPS-II NC 184 185	Populational	B-cell NHL and Multiple myeloma<br>Total cases 375	Q4(40.94+µg/L)RR1.52; CI95%(1.02. 2.25); p 0.08	[5]
20 741	Occupational exposure	Lung Cancer<br>Total cases 53	Q3(1.4-1.9µmol/L)OR1.7;CI95%(0.7-3.9)	[6]
4573	Populational	Total cases 541 Any cancers	Q4HR 1.51;CI95%(1.07.2.12);p 0.04	[7]
367	Populational	Total cases 100 Lung cancer	OR0.98;CI(0.81-1.19);p 0.834	[8]
1725	Populational	Total cases<br>585<br>Gastroesophageal cancers	Q3 fingernails levels OR2.32;CI(1.58-3.46)	[9]
EPIC-Italy 300	Populational	Total cases 150<br>Breast cancer	Tertile2(1.5-2.3µg/L) OR0.74;CI(0.23-2.34);p0.603	[10]
EPIC-Spain<br>600	Populational	Total cases 300<br>Breast cancer	Q3(0.3-0.5ng/ml) OR1.3;CI(0.95-1.77);p 0.1	[11]

References

1. Kelly, R. S., Lundh, T., Porta, M., Bergdahl, I. A., Palli, D., Johansson, A. S., Botsivali, M., Vineis, P., Vermeulen, R., Kyrtopoulos, S. A., Chadeau-Hyam, M., & EnviroGenoMarkersProject Consortium (2013). Blood erythrocyte concentrations of cadmium and lead and the risk of B-cell non-Hodgkin's lymphoma and multiple myeloma: a nested case-control study. *PloS one*, *8*(11), e81892. https://doi.org/10.1371/journal.pone.0081892
2. Steenland K, Barry V, Anttila A, Sallmen M, Mueller W, Ritchie P, McElvenny DM, Straif K. Cancer incidence among workers with blood lead measurements in two countries. Occup Environ Med. 2019 Sep;76(9):603-610. doi: 10.1136/oemed-2019-105786. Epub 2019 Jul 11. PMID: 31296664.
3. Wang M, Yu Q. Association between blood heavy metal concentrations and skin cancer in the National Health and Nutrition Examination Survey, 2011-2018. Environ Sci Pollut Res Int. 2023 Oct;30(50):108681-108693. doi: 10.1007/s11356-023-29674-4. Epub 2023 Sep 26. PMID: 37751003.
4. Cao HM, Yang YZ, Huang BY, Zhang Y, Wu Y, Wan Z, Ma L. A cross-sectional study of the association between heavy metals and pan-cancers associated with sex hormones in NHANES 1999-2018. Environ Sci Pollut Res Int. 2023 May;30(21):61005-61017. doi: 10.1007/s11356-023-26828-2. Epub 2023 Apr 12. PMID: 37046159.
5. Deubler EL, Gapstur SM, Diver WR, Gaudet MM, Hodge JM, Stevens VL, McCullough ML, Haines LG, Levine KE, Teras LR. Erythrocyte levels of cadmium and lead and risk of B-cell non-Hodgkin lymphoma and multiple myeloma. Int J Cancer. 2020 Dec 1;147(11):3110-3118. doi: 10.1002/ijc.33136. Epub 2020 Jun 25. PMID: 32506449.
6. Anttila A, Heikkilä P, Pukkala E, Nykyri E, Kauppinen T, Hernberg S, Hemminki K. Excess lung cancer among workers exposed to lead. Scand J Work Environ Health. 1995 Dec;21(6):460-9. doi: 10.5271/sjweh.62. PMID: 8824752.
7. Li Z, Long T, Wang R, Feng Y, Hu H, Xu Y, Wei Y, Wang F, Guo H, Zhang X, He M. Plasma metals and cancer incidence in patients with type 2 diabetes. Sci Total Environ. 2021 Mar 1;758:143616. doi: 10.1016/j.scitotenv.2020.143616. Epub 2020 Nov 12. PMID: 33218808.
8. Unrine JM, Slone SA, Sanderson W, Johnson N, Durbin EB, Shrestha S, Hahn EJ, Feltner F, Huang B, Christian WJ, Mellon I, Orren DK, Arnold SM. A case-control study of trace-element status and lung cancer in Appalachian Kentucky. PLoS One. 2019 Feb 27;14(2):e0212340. doi: 10.1371/journal.pone.0212340. PMID: 30811496; PMCID: PMC6392268.

9. Zhang T, Yin X, Yang X, Yuan Z, Wu Q, Jin L, Chen X, Lu M, Ye W. Trace elements in hair or fingernail and gastroesophageal cancers: results from a population-based case-control study. J Expo Sci Environ Epidemiol. 2023 Nov;33(6):933-944. doi: 10.1038/s41370-023-00528-y. Epub 2023 Feb 24. PMID: 36828865.

10. Caini S, Cozzolino F, Saieva C, Aprea MC, De Bonfioli Cavalcabo' N, Ermini I, Assedi M, Biagiotti D, Trane C, Facchini L, Bendinelli B, Palli D, Masala G. Serum heavy metals and breast cancer risk: A case-control study nested in the Florence cohort of the EPIC (European Prospective Investigation into Cancer and nutrition) study. Sci Total Environ. 2023 Feb 25;861:160568. doi: 10.1016/j.scitotenv.2022.160568. Epub 2022 Dec 1. PMID: 36464039.

11. Fernández-Martínez NF, Rodríguez-Barranco M, Huerta JM, Gil F, Olmedo P, Molina-Montes E, Guevara M, Zamora-Ros R, Jiménez-Zabala A, Colorado-Yohar SM, Ardanaz E, Bonet C, Amiano P, Chirlaque MD, Pérez-Gómez B, Jiménez-Moleón JJ, Martín-Jiménez M, de Santiago E, Sánchez MJ. Breast cancer risk for the joint exposure to metals and metalloids in women: Results from the EPIC-Spain cohort. Sci Total Environ. 2024 Feb 20;912:168816. doi: 10.1016/j.scitotenv.2023.168816. Epub 2023 Nov 28. PMID: 38036124.
